# VH-CH1 switch region-inserting multispecific antibody designs and their efficacy against SARS-CoV-2 in vitro and in vivo

**DOI:** 10.1038/s41421-023-00616-1

**Published:** 2023-11-11

**Authors:** Lili Wu, Yue Gao, Dandan Yu, Sheng Liu, Runchu Zhao, Dezhi Liu, Ling Xu, Honghui Liu, Xiaoyun Wang, Jianxun Qi, Yan Chai, Liya Wei, Yong-Gang Yao, George F. Gao, Qihui Wang

**Affiliations:** 1 CAS Key Laboratory of Pathogen Microbiology and Immunology, Institute of Microbiology, Chinese Academy of Sciences, Beijing, China; 2 College of Life Science, Hebei University, Baoding, Hebei China; 3 Key Laboratory of Animal Models and Human Disease Mechanisms of the Chinese Academy of Sciences, and KIZ-CUHK Joint Laboratory of Bioresources and Molecular Research in Common Diseases, Kunming Institute of Zoology, Chinese Academy of Sciences, Kunming, Yunnan China; 4 Kunming National High-level Biosafety Research Center for Non-Human Primates, Center for Biosafety Mega-Science, Kunming Institute of Zoology, Chinese Academy of Sciences, Kunming, Yunnan China; 5 Cryo-EM Center, Department of Biology, Southern University of Science and Technology, Shenzhen, Guangdong China; 6 Institute of Physical Science and Information, Anhui University, Hefei, Anhui China; 7 University of the Chinese Academy of Sciences, Beijing, China; 8 National Resource Center for Non-Human Primates, National Research Facility for Phenotypic & Genetic Analysis of Model Animals (Primate Facility), Kunming Institute of Zoology, Chinese Academy of Sciences, Kunming, Yunnan China; 9 Chinese Center for Disease Control and Prevention, Beijing, China

**Keywords:** Molecular biology, Cell biology

Dear Editor,

To combat SARS-CoV-2 infection, numerous monoclonal antibodies have been developed. However, the virus’s continuous variation has rendered most antibodies ineffective^1,2^. Although there are broad-spectrum antibodies targeting conserved epitopes on the spike (S) proteins, their neutralizing potency is generally moderate^1–3^, constraining further development. Rational design of multispecific antibodies could enhance the potency compared to parental counterparts^4^. Several strategies have been explored to counter SARS-CoV-2, like the IgG-scFv format of bsAb15^5^ and linker-connected bn03^6^, but their configurations are artificially engineered. Here, utilizing the natural VH-CH1 switch region-inserting scaffold found in malaria-exposed individuals^7^, we designed multiple bispecific (bsAbs) and trispecific (tsAbs) antibodies by combining the antibodies recognizing conserved epitopes on SARS-CoV-2 S but exhibiting moderate effectiveness.

Initially, by screening a previous nanobody library^8^, we identified a SARS-CoV-2 receptor-binding domain (RBD)-targeting nanobody R211. Binding assays revealed that R211 potently bound to all tested RBDs from SARS-CoV-2 and its variants, but its activities decreased in binding Omicron sub-variants (Supplementary Fig. S1). Pseudovirus-based neutralization results indicated that R211 showed broad but moderate potency against SARS-CoV-2 variants with half-maximal inhibitory concentration (IC_50_) values around 0.1 μg/mL, and the activity further decreased when neutralizing Omicron sub-variants (Supplementary Fig. S2). Flow cytometry-based assays further indicated that R211 can broadly bind to all tested sarbecovirus S proteins, though its ability was lower than our previously identified broad-spectrum antibodies IMCAS74 (namely 74) and S102 (Supplementary Fig. S3). Competition-binding assays indicated that, among the antibodies targeting eight epitope classes on RBD^2^, R211 could compete with ADI-56046 (RBD-3), C022 (RBD-6) and H014 (RBD-7) (Supplementary Fig. S4), suggesting that R211 likely recognizes a cryptic epitope. Cryo-EM structure of R211 and SARS-CoV-2 S further revealed that R211 recognized the RBD-7 epitope (Supplementary Figs. S5, S6 and Table S1), and the binding sites were highly conserved among sarbecoviruses (Supplementary Fig. S6d). Detailed analysis indicated that S371L/F mutation in Omicron may break the hydrogen-bond interaction with R211 (Supplementary Fig. S6c), thereby weakening the binding.

Based on the broad breadth but moderate efficacy of R211 and three previous antibodies (74, S102 and R14), we supposed that combining these nonoverlapping antibodies may improve the neutralizing potency against SARS-CoV-2 variants (Fig. 1a). 74 recognizes a conserved epitope on the RBD as S2H97^9^, showing resistance to escape by SARS-CoV-2 variants^2^. S102 targets the conserved stem-helix region of the S2 and exhibits pan-sarbecovirus neutralization^3^. R14 recognizes the receptor-binding motif and displays varied neutralization against SARS-CoV-2 variants^8^. Particularly, R211, S102 and R14 are nanobodies, which are easily manipulated. Therefore, we utilized the natural VH-CH1-inserting antibody format to design bsAbs by inserting nanobodies into the switch region between VH and CH1 of 74^7^, resulting in three bsAbs (R211-74, S102-74 and R14-74) (Fig. 1b). Simultaneously, we also designed three other bsAbs (74-R211, 74-S102 and 74-R14) for comparison based on the IgG-Fv format by fusing nanobodies to the CH3 of 74 (Fig. 1b).

**Fig. 1:**
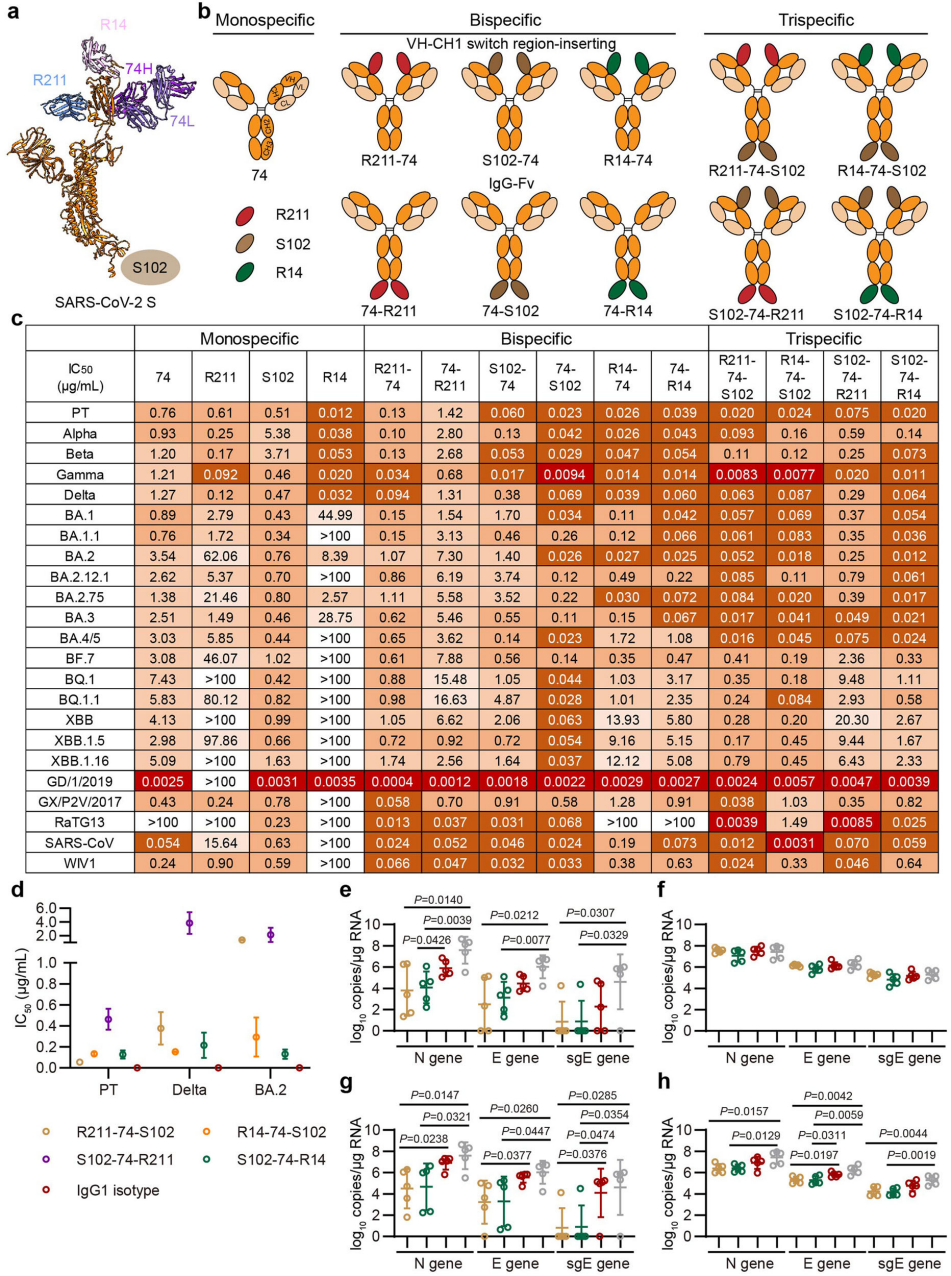
Design and efficacy of multispecific antibodies. **a** Epitopes of R211, 74, R14 and S102 on SARS-CoV-2 S protein with S102 shown schematically due to its unrevealed structure. **b** Schematic representation of designed multispecific antibodies. **c** Neutralization of multispecific antibodies against pseudotyped SARS-CoV-2 and other sarbecoviruses. The assay was independently repeated twice with two replicates (*n* = 2). Mean IC_50_s from two independent experiments are shown. PT, SARS-CoV-2 prototype strain. **d** Neutralization of trispecific antibodies against live SARS-CoV-2 PT, Delta and BA.2. The assay was repeated twice with at least four replicates (*n* ≥ 4). Mean IC_50_s from two independent experiments are shown, with different antibodies represented by indicated circles. **e**, **f** Syrian hamsters (*n* = 5 per group) were prophylactically treated intraperitoneally (i.p.) with indicated antibody or PBS 6 h before intranasal (i.n.) challenge with BA.2. Viral titers of the N, E and subgenomic E (sgE) genes in lung (**e**) and nasal turbinate (**f**) 3 days post-infection (dpi) were detected. Antibodies are represented by different circles as in **d**, with gray circle indicating PBS. **g**, **h** Syrian hamsters (*n* = 5 per group) were therapeutically treated i.p. with the indicated antibody or PBS 6 h after i.n. challenge with BA.2. Viral titers of the N, E and sgE genes in lung (**g**) and nasal turbinate (**h**) 3dpi were detected. Antibodies are represented by different circles as in **e**. The *P* values in **e**–**h** were analyzed using unpaired two-tailed *t*-test.

Size-exclusion chromatography and SDS-PAGE profiles indicated that both formats of bsAbs can assemble correctly, exemplified by S102-74 and 74-S102 (Supplementary Fig. S7). To evaluate the efficacy of these bsAbs, we performed pseudovirus-based neutralization assays (Fig. 1c; Supplementary Fig. S8). We first evaluated their potency against SARS-CoV-2 prototype (PT) and the variants until BA.4/5. Regarding the VH-CH1-inserting format, compared to 74, R211-74 showed 3.0–35.9-fold increase against SARS-CoV-2 variants except BA.2.75; S102-74 exhibited 2.5–71.4-fold enhancement against Alpha, Beta, Gamma, Delta, BA.2, BA.3 and BA.4/5 and similar potency against other variants; and R14-74 displayed 5.3–130.1-fold enhancement against SARS-CoV-2 variants before BA.4/5 and slight increase against BA.4/5. While regarding the IgG-Fv format, 74-R211 showed similar or slightly decreased neutralization against SARS-CoV-2 variants compared to 74 and was lower than R211-74; 74-S102 displayed 2.9–135.4-fold enhancement against SARS-CoV-2 variants compared to 74 and was higher than S102-74; and 74-R14 showed similar potency to R14-74 and both were higher than 74. Notably, among these six bsAbs, 74-S102 showed the strongest potency against BA.4/5 (Fig. 1c).

Based on the effectiveness of these two bispecific formats, namely 74-S102 and S102-74, we further inserted R211 or R14 into either bsAb to design tsAbs, including R211-74-S102, R14-74-S102, S102-74-R211 and S102-74-R14 (Fig. 1b). The four tsAbs also assembled correctly, exemplified by R14-74-S102 and S102-74-R14 (Supplementary Fig. S7). Neutralization assays indicated that compared to 74-S102, R211-74-S102 and R14-74-S102 exhibited slightly increased or similar potency against SARS-CoV-2 variants including BA.4/5 (Fig. 1c; Supplementary Fig. S8). S102-74-R211 was weaker than R211-74-S102 but 4.2–112.1-fold better than 74-R211 against SARS-CoV-2 variants. S102-74-R14 was similar to R14-74-S102 and displayed comparable or slightly enhanced ability against SARS-CoV-2 variants compared to 74-R14.

Because of the potent neutralizing activities of tsAbs against pseudotyped SARS-CoV-2 variants, we also tested their potency against live SARS-CoV-2 virus. R211-74-S102, R14-74-S102 and S102-74-R14 showed good potencies against SARS-CoV-2 PT, Delta and BA.2, with IC_50_ values below 0.5 μg/mL, except R211-74-S102 against BA.2 (Fig. 1d). R14-74-S102 and S102-74-R14 showed comparable activities against these viruses. In contrast, S102-74-R211 displayed weak neutralization against these viruses, consistent with the pseudovirus results (Fig. 1c). According to the in vitro potencies, we further selected R211-74-S102 and S102-74-R14 to assess their in vivo prophylactic and therapeutic activities (Fig. 1e–h). In the prophylactic groups, hamsters that received 15 mg/kg of R211-74-S102 or S102-74-R14 showed a significant 2–4-log reduced viral titers in the lung compared to the control groups (Fig. 1e). Notably, four of the five hamsters in both R211-74-S102 and S102-74-R14 groups exhibited undetectable subgenomic RNA of the E gene (sgE) in the lung. However, no significant improvement in viral titers in the nasal turbinate was observed (Fig. 1f). In the therapeutic groups, the two antibodies not only reduced viral titers in the lung (Fig. 1g) but also in the nasal turbinate (Fig. 1h). Consistent with the reduction of viral titers in the prophylactic groups, hamsters treated therapeutically with R211-74-S102 or S102-74-R14 exhibited undetectable sgE in the lungs of four of the five individuals. Overall, these results indicated that the two tsAbs can effectively prevent SARS-CoV-2 infections in the lungs when used prophylactically or therapeutically.

With the continuous evolution of Omicron, we assessed the neutralizing potency of the bsAbs and tsAbs against recent BF.7, BQ and XBB. Similarly, compared to 74, R211-74, S102-74 and 74-S102 still showed increased potency against these sub-variants, particularly 74-S102, which exhibited 55.0–205.5-fold enhancement against BQ and XBB with IC_50_ below 0.1 μg/mL (Fig. 1c; Supplementary Fig. S8). 74-R211 showed similar or slightly decreased potency against these sub-variants compared to 74. However, although R14-74 and 74-R14 displayed enhanced activities against BF.7 and BQ, they showed decreased potency against XBB. S102-74-R14 showed enhanced potencies against BF.7, BQ and XBB compared to 74-R14. S102-74-R211 showed increased potencies against BF.7 and BQ but decreased ability against XBB compared to 74-R211. Unexpectedly, compared to 74-S102, R211-74-S102 and R14-74-S102 exhibited decreased capabilities against BF.7, BQ and XBB. Additionally, we also assessed the efficacies of cocktail of 74, S102 and R211 and cocktail of 74, S102 and R14 against SARS-CoV-2 PT, Beta, BA.1, BA.2, BA.5 and XBB, which represent different serotypes according to our recently published data^10^. The results revealed that the efficacy of the cocktail of 74, R211 and S102 was generally weaker than that of S102-74-R211 and especially R211-74-S102 (Fig. 1c; Supplementary Fig. S9). Similarly, the efficacy of the cocktail of 74, R14 and S102 was also weaker than that of R14-74-S102 and S102-74-R14, but they were comparable when neutralizing PT and Beta, which may be due to the potent neutralization of R14.

Moreover, when neutralizing other sarbecoviruses, R211-74, 74-R211, S102-74 and 74-S102, especially R211-74, showed increased ability, whereas R14-74 and 74-R14 displayed comparable or slightly decreased activities, compared to 74 (Fig. 1c; Supplementary Fig. S8). Compared to 74-S102, R211-74-S102 showed enhanced potency, but R14-74-S102 displayed decreased activity. S102-74-R211 and S102-74-R14 exhibited similar or slightly increased abilities compared to 74-R211 and 74-R14, respectively. Notably, among these ten multispecific antibodies, R211-74-S102 displayed the highest potency against tested sarbecoviruses.

Antibody engineering plays a significant role in antibody therapy. Here, we explored a strategy mimicking the natural VH-CH1-inserting antibody identified in malaria-exposed individuals to design multispecific antibodies^7^, which exhibited significant enhancements against SARS-CoV-2 variants and other sarbecovirues in vitro. In hamsters, tsAbs reduced viral titers in lung when used prophylactically or therapeutically. However, the difference observed in nasal turbinate between the prophylactic and therapeutic groups may be related to the limited antibody diffusion from circulation to nasal turbinate and the antibody pharmacokinetics. Our findings manifested the improved potency of these multispecific antibodies, suggesting potential antibody candidates against SARS-CoV-2 variants, as well as demonstrating the power of this natural scaffold for future antibody engineering.

### Supplementary information

Supplementary Information

## Data Availability

Cryo-EM density map and atomic coordinates have been deposited in the Electron Microscopy Data Bank and Protein Data Bank under the codes EMD-36775 and 8K0N, respectively.
