# Supplementary material for: VH-CH1 switch region-inserting multispecific antibody designs and their efficacy against SARS-CoV-2 in vitro and in vivo
Source: Cell Discov. 2023 Nov 11;9:113. doi: 10.1038/s41421-023-00616-1 (PMC10640590; doi:10.1038/s41421-023-00616-1)
Supplement: Supplementary file 1 — Supplementary Information [file 41421_2023_616_MOESM1_ESM.pdf]

Supplementary information

Fig. S1

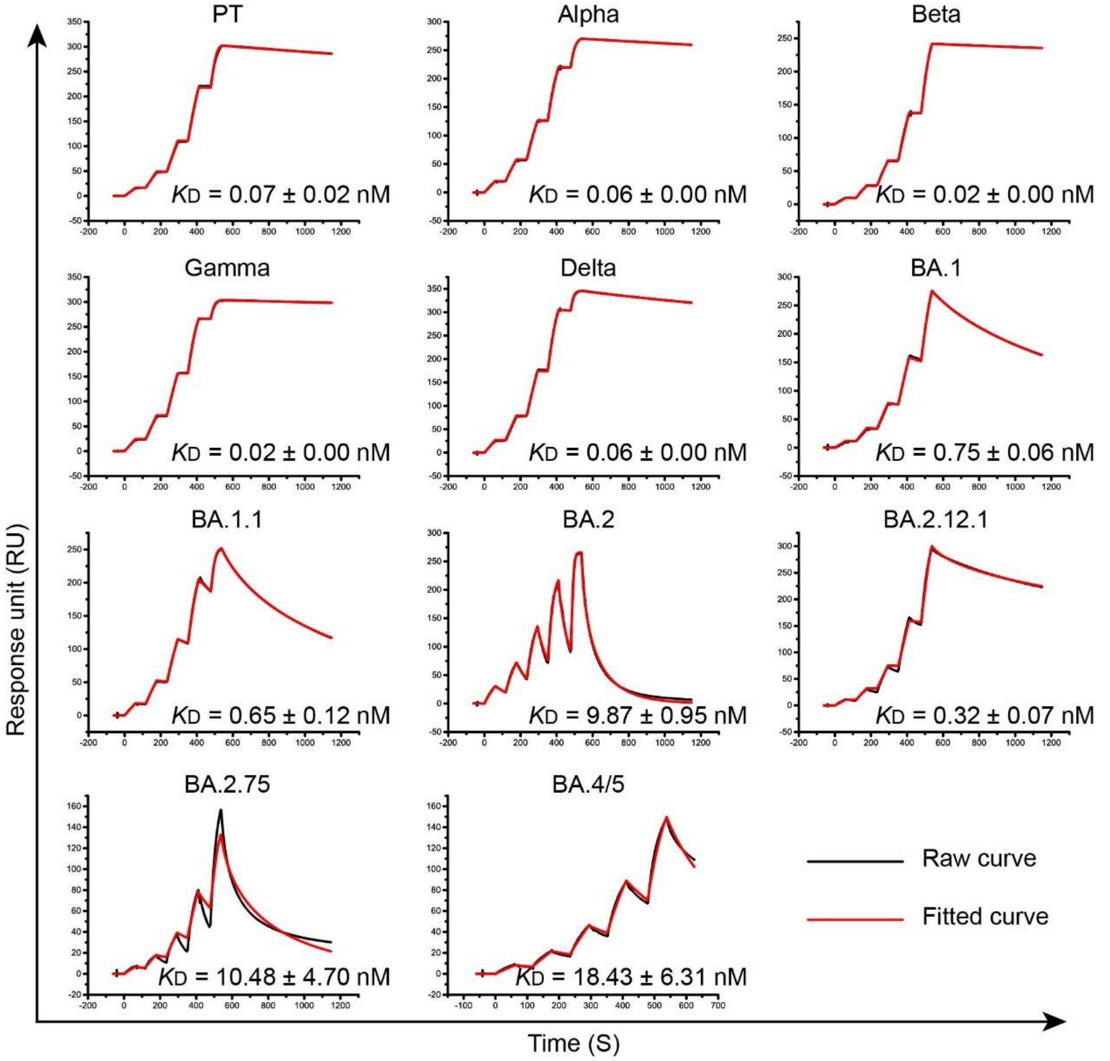

Supplementary Fig. S1 Binding of R211 to RBDs from SARS-CoV-2 prototype (PT) and variants of concern (VOCs) tested by surface plasmon resonance (SPR) assays. The assay was repeated three times. The equilibrium dissociation constant (K_D) values were the mean ± standard deviation (SD) of three independent experiments. The raw and fitted binding curves are shown as black and red lines, respectively. One representative run is shown of three independent experiments.

Fig. S2

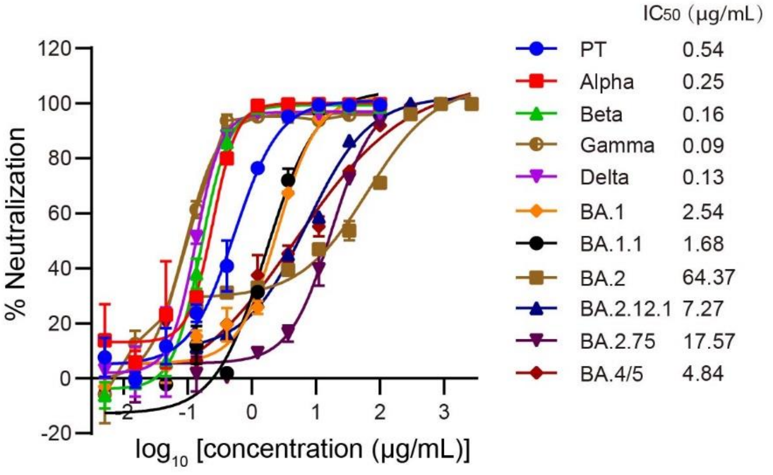

Supplementary Fig. S2 Neutralizing activity of R211 against pseudotyped SARS-CoV-2 PT and VOCs. The assay was performed twice with two replicates (n=2) at each time. Representative results of two independent experiments are shown.

Fig. S3

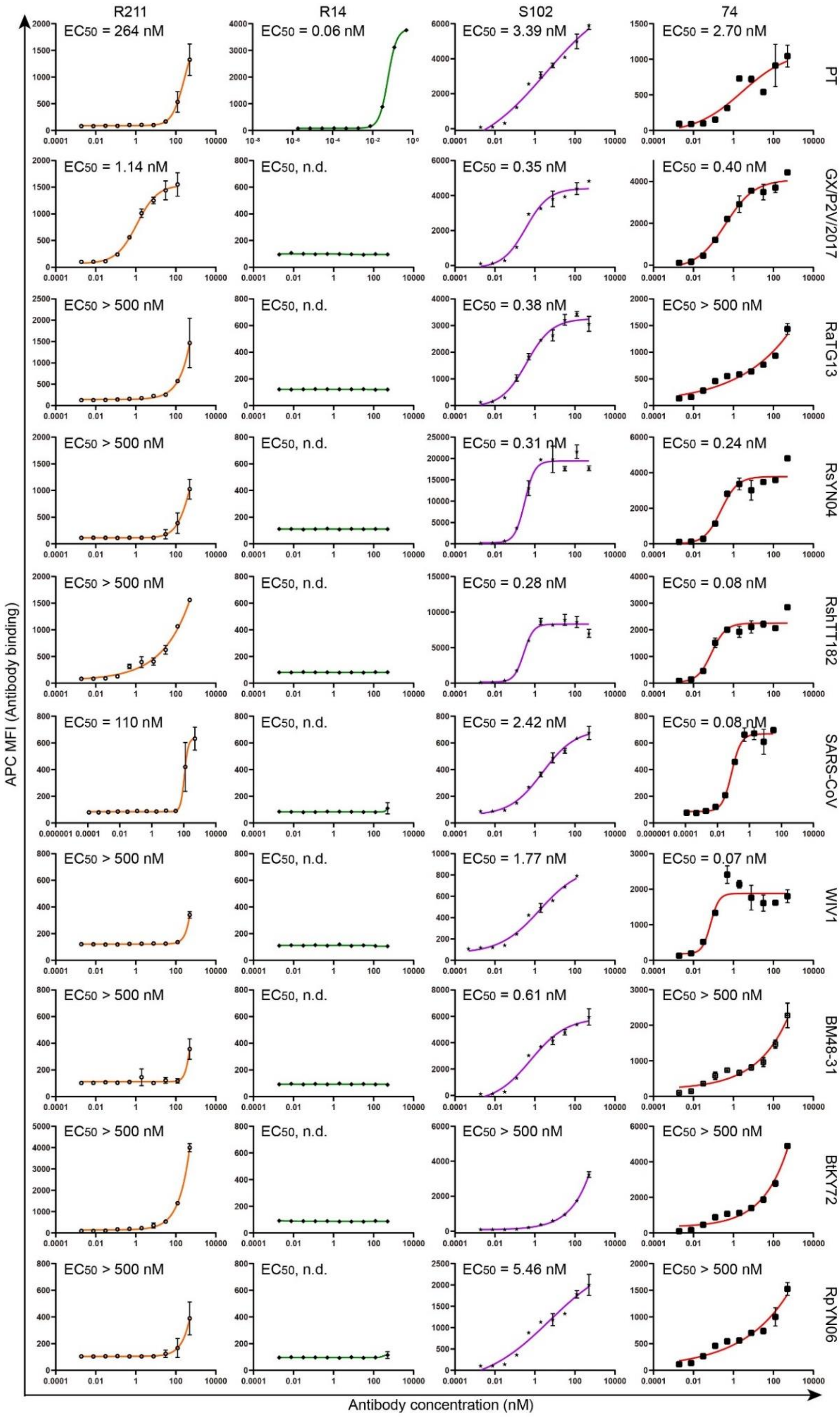
R211
R14
S102
74
PT
GX/P2V/2017
RaTG13
RsYN04
RshTT182
SARS-CoV
WIV1
BM48-31
BtKY72
RpYN06
EC50 = 264 nM
EC50 = 0.06 nM
EC50 = 3.39 nM
EC50 = 2.70 nM
EC50 = 1.14 nM
EC50, n.d.
EC50 = 0.35 nM
EC50 = 0.40 nM
EC50 > 500 nM
EC50, n.d.
EC50 = 0.38 nM
EC50 > 500 nM
EC50 > 500 nM
EC50, n.d.
EC50 = 0.31 nM
EC50 = 0.24 nM
EC50 > 500 nM
EC50, n.d.
EC50 = 0.28 nM
EC50 = 0.08 nM
EC50 = 110 nM
EC50, n.d.
EC50 = 2.42 nM
EC50 = 0.08 nM
EC50 > 500 nM
EC50, n.d.
EC50 = 1.77 nM
EC50 = 0.07 nM
EC50 > 500 nM
EC50, n.d.
EC50 = 0.61 nM
EC50 > 500 nM
EC50 > 500 nM
EC50, n.d.
EC50 > 500 nM
EC50 > 500 nM
EC50 > 500 nM
EC50, n.d.
EC50 = 5.46 nM
EC50 > 500 nM
APC MFI (Antibody binding)
Antibody concentration (nM)

Supplementary Fig. S3 Flow cytometry-based binding of R211, R14, S102 and 74 to spike (S) proteins of sarbecoviruses belonging to four clades. SARS-CoV-2 PT, GX/P2V/2017, RaTG13, RsYN04 and RsTTh182 belong to SARS-CoV-2 clade, SARS-CoV and WIV1 belong to SARS-CoV clade, BM48-31 and BtKY72 belong to Asia and Europe clade, and RpYN06 belongs to non-ACE2 binding clade. The assay was repeated twice with two technical replicates (*n*=2) at each time. Representative results of two independent experiments are shown. n.d, not determined.

Fig. S4

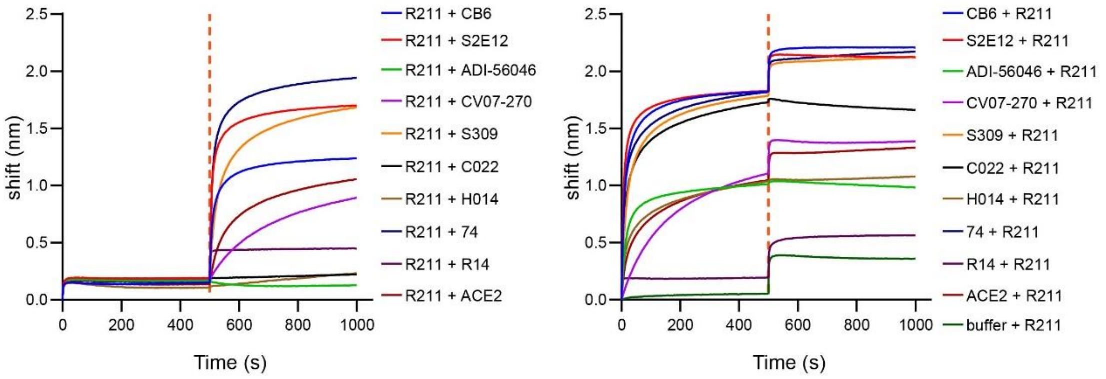

CB6 (RBD-1)	S2E12 (RBD-2)	ADI-56046 (RBD-3)	CV07-270 (RBD-4)
S309 (RBD-5)	C022 (RBD-6)	H014 (RBD-7)	74 (RBD-8)

Supplementary Fig. S4 Competitive binding of R211 and monoclonal antibodies belonging to eight epitope classes on SARS-CoV-2 RBD, as measured by Octet RED96. The assay was repeated twice. Shown data are one representative result.

Fig. S5

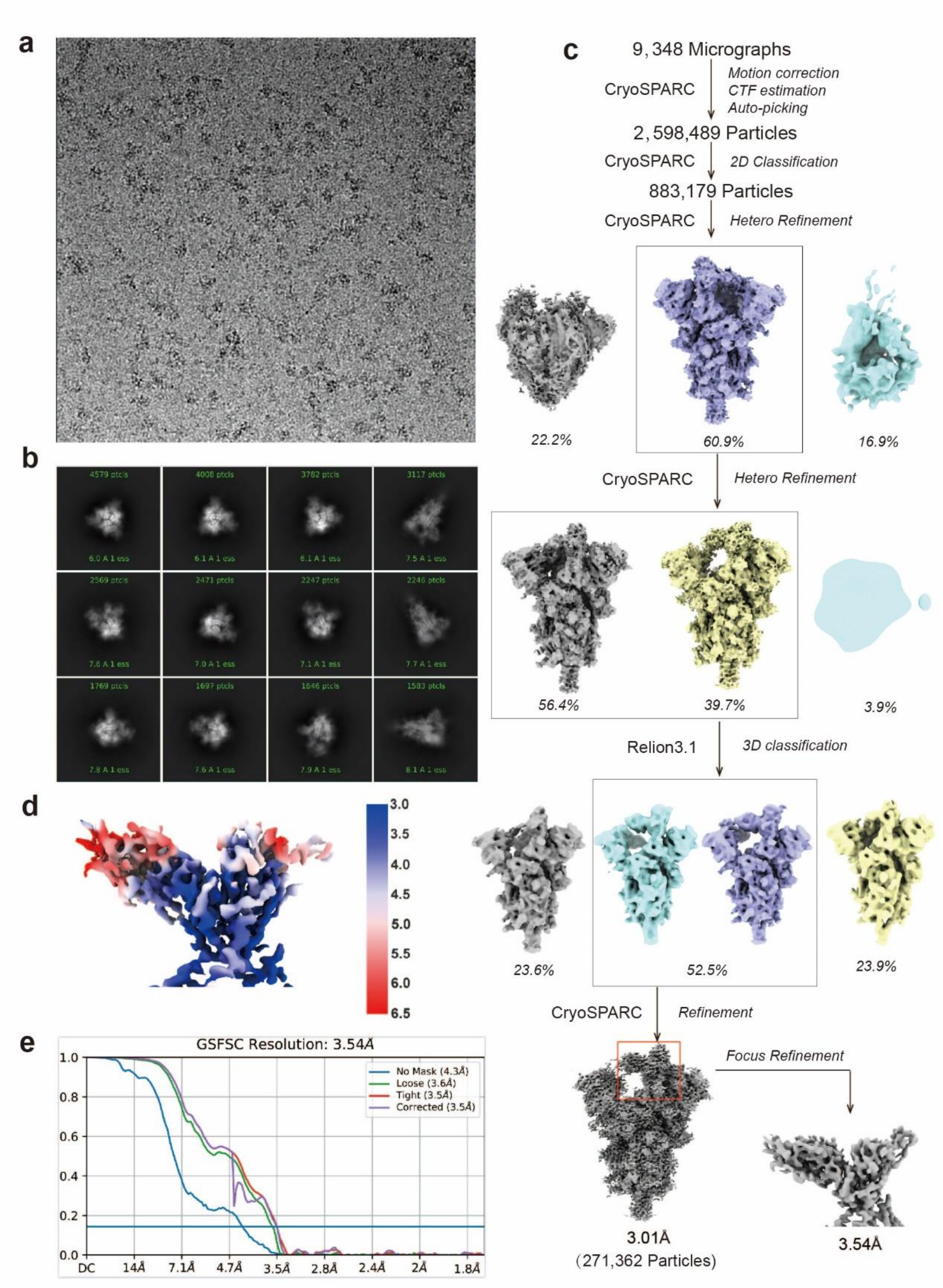

Supplementary Fig. S5 Flow chart of single-particle analysis of the R211 in complex with SARS-CoV-2 S. a Representative cryo-EM micrograph of the R211/SARS-CoV-2 S. **b** 2D class average images of the R211/SARS-CoV-2 S. **c** A brief

workflow of cryo-EM image processing and reconstruction. **d** Cryo-EM map of the R211/SARS-CoV-2 S, colored by local resolution (Å). **e** The Fourier shell correlation (FSC) curve for reconstruction.

Fig. S6

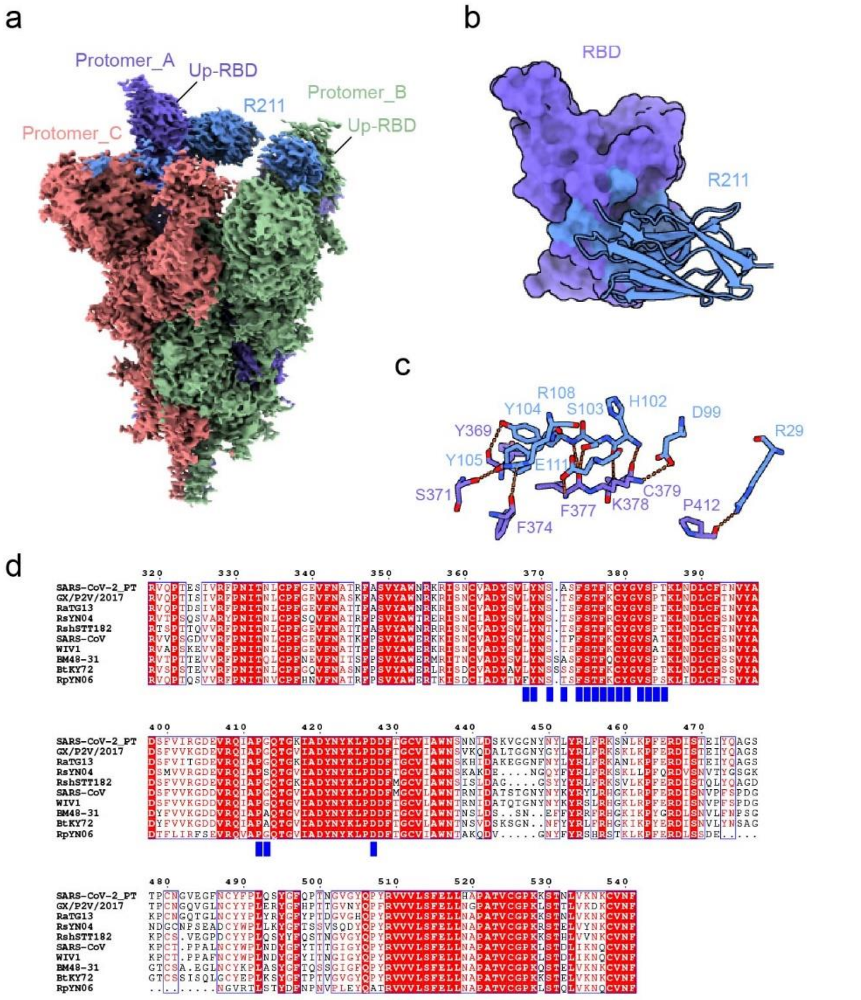

Supplementary Fig. S6 Cryo-EM structure of R211 in complex with SARS-CoV-2 RBD. **a** Cryo-EM map of R211 in complex with SARS-CoV-2 S at a 3.01 Å global resolution. **b** The complex structure of R211 and SARS-CoV-2 RBD at 3.5 Å resolution after local refinement. The footprint of R211 on RBD was displayed. **c** Detailed interaction between R211 and RBD. Dashed lines represent hydrogen bonds or salt bridges. **d** The sequence conservation of the R211-binding epitope in the sarbecoviruses. The binding sites of R211 on SARS-CoV-2 RBD were indicated in blue rectangles.

Fig. S7

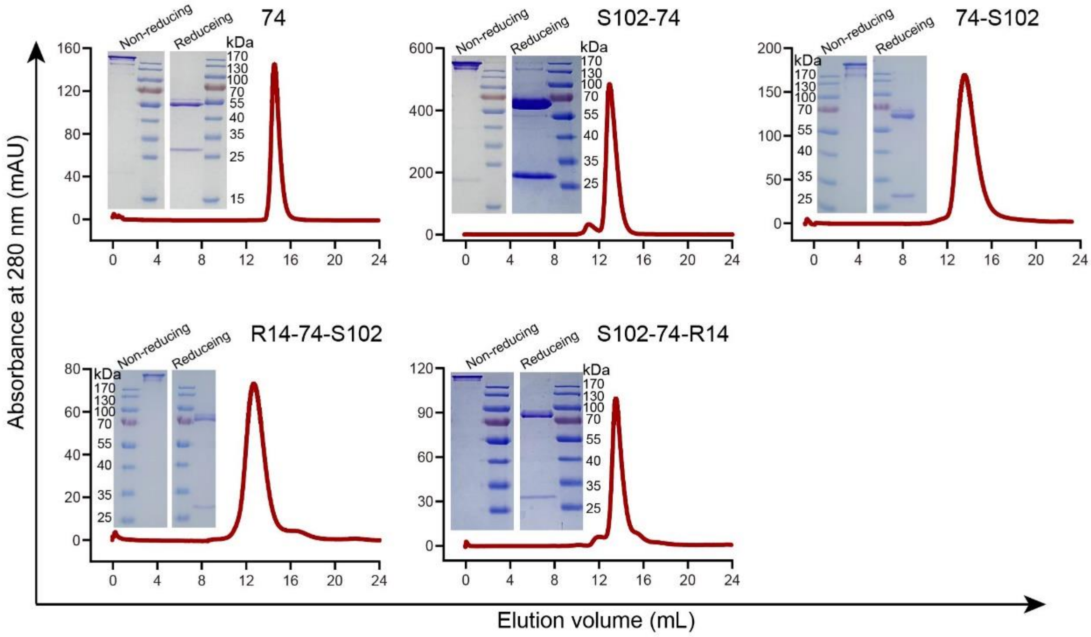

Supplementary Fig. S7 Size-exclusion chromatography analysis and SDS–PAGE profiles (non-reducing and reducing) of multispecific antibodies. The chromatography analysis was measured using Superdex 200 Increase 10/300 GL columns (GE Healthcare).

Fig. S8

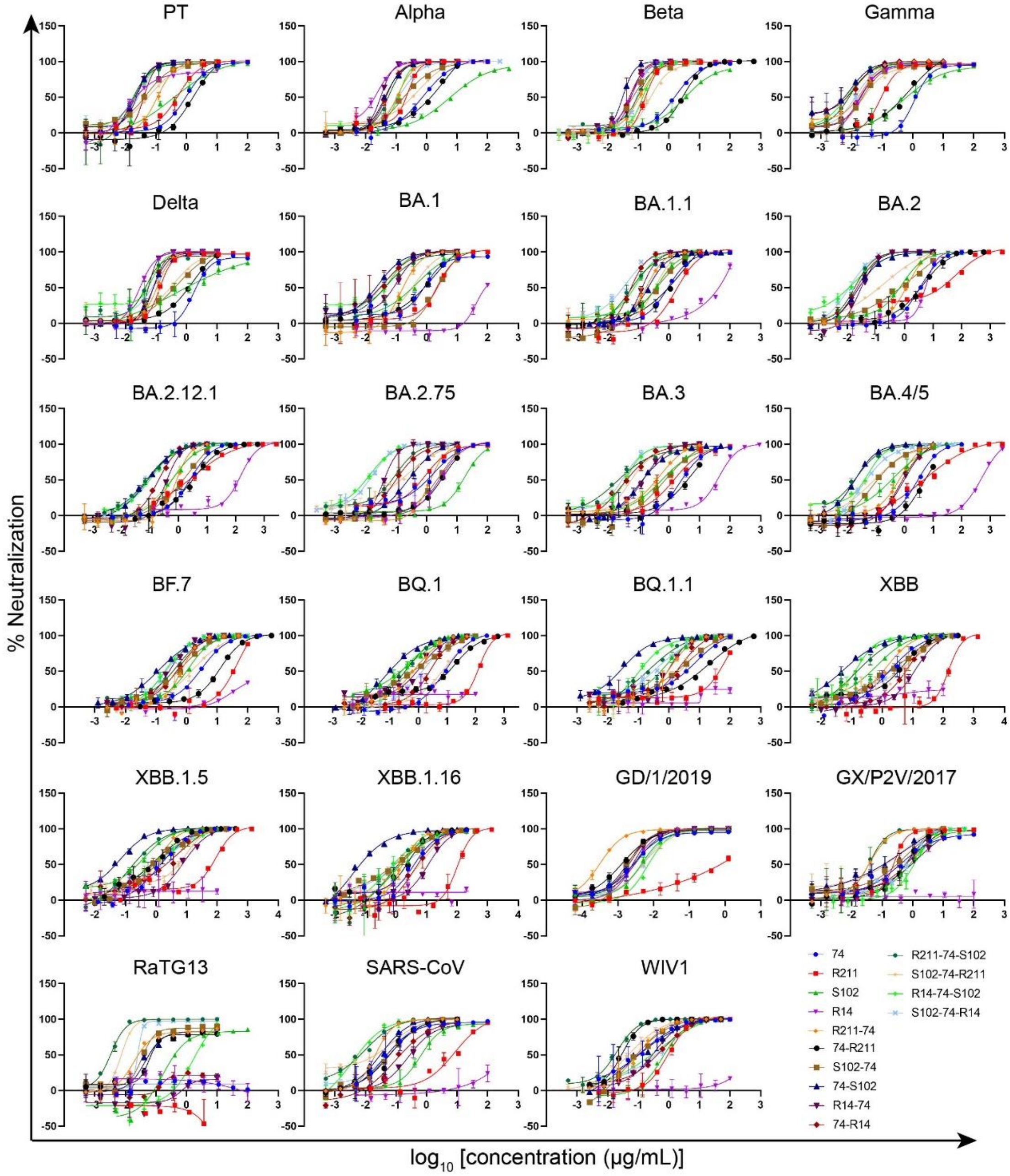

Supplementary Fig. S8 Neutralization curves of 74, R211, S102, R14 and their multispecific antibodies against pseudotyped SARS-CoV-2 VOCs and other sarbecoviruses. The neutralization curves shown here are one representative result of two independent experiments.

Fig. S9

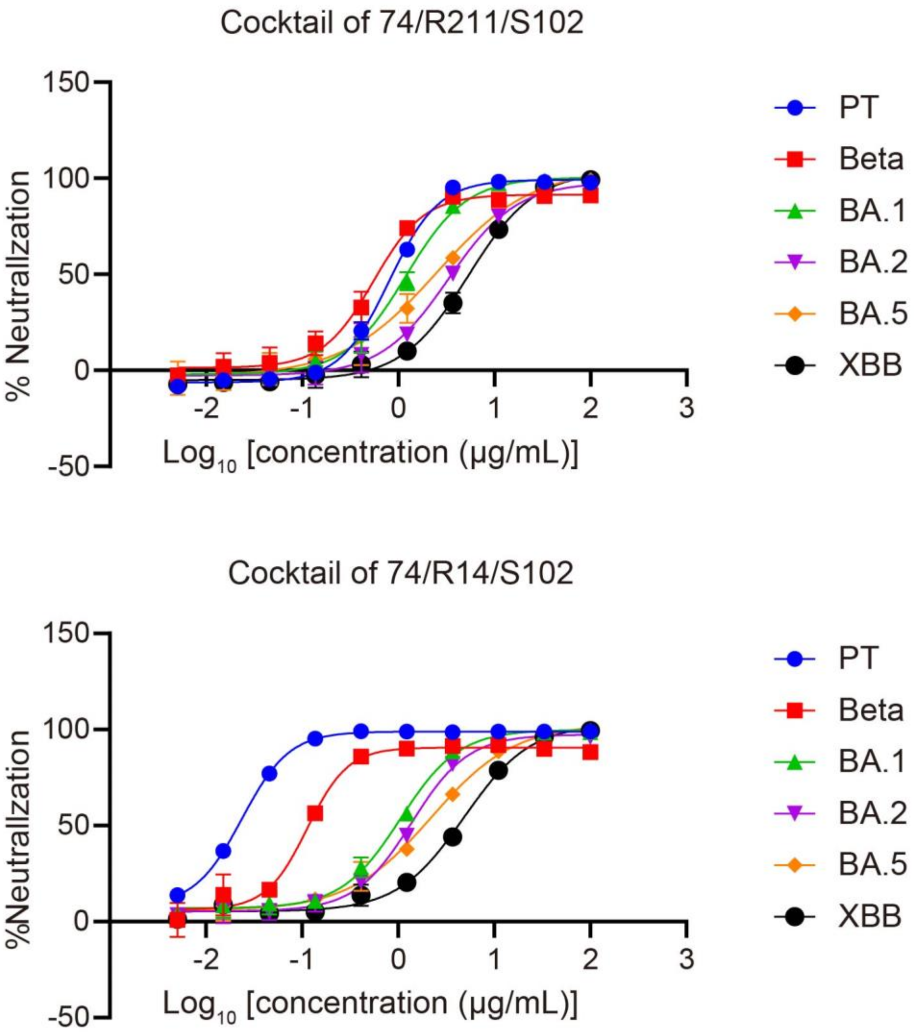

IC_{50} (μg/mL)	**PT**	**Beta**	**BA.1**	**BA.2**	**BA.5**	**XBB**
**cocktail of 74/R211/S102**	0.81	0.56	1.17	3.29	2.38	5.16
**cocktail of 74/R14/S102**	0.026	0.10	1.05	1.37	2.30	4.86

Supplementary Fig. S9 Neutralization of cocktail of 74, S102 and R211 or R14 against several pseudotyped SARS-CoV-2 VOCs. The neutralization curves shown here are one representative result of two independent experiments. The neutralizing activities (IC_{50}) are the mean of two independent experiments.

Supplementary Table S1 Cryo-EM data collection, refinement and validation statistics

	R211/SARS-CoV-2 RBD
**Data collection and processing**	
Magnification	105k
Voltage (kV)	300
Electron exposure (e^-/Å^2)	50
Defocus range (μm)	-1.2 to -2.2
Pixel size (Å)	0.84
Symmetry imposed	C1
Initial particle images (no.)	2,598,489
Final particle images (no.)	271,362
Map resolution (Å)	3.54
FSC threshold	0.143
Map resolution range (Å)	3.0-6.0
**Refinement**	
Initial model used (PDB code)	6M0J
Model resolution (Å)	3.54
Map sharpening *B* factor (Å^2)	-110.4
Model composition	
Non-hydrogen atoms	2513
Protein residues	317
Ligands	1
R.m.s. deviations	
Bond lengths (Å)	0.005
Bond angles (°)	0.982
Validation	
MolProbity score	1.96
Clashscore	8.60
Poor rotamers (%)	1.13
Ramachandran plot	
Favored (%)	95.53
Allowed (%)	4.47
Disallowed (%)	0.00

Materials and methods

Cells, viruses and animals

Vero E6 (ATCC, CRL-1586), HEK293T (ATCC, CRL-3216), BHK-21(ATCC, CCL-10) and HEK293T-hACE2 (Genewiz®) were grown at 37 °C in Dulbecco's modified Eagle's medium (DMEM) supplemented with 10% fetal bovine serum (FBS). Freestyle 293F cells were cultured in SMM 293-TII medium at 37 °C in a shaker with 5% CO_2. The SARS-CoV-2 PT (Accession No. NMDCN0000HUI) was kindly provided by Guangdong Provincial Center for Disease Control and Prevention (Guangdong, China). The SARS-CoV-2 Delta (Accession No. NMDC60042793) and Omicron BA.2 (Accession No. NMDC60046377) strains were isolated in the Biosafety Level 3 (BSL3) facility of Kunming Institute of Zoology, Chinese Academy of Sciences (CAS). All virus strains were propagated by using the same protocol described in our previous studies[1,2]. Specific pathogen-free (SPF) male Syrian hamsters (3–4 weeks) were purchased from Vital River (Beijing, China). All animal experiments in this study were approved by the Institutional Animal Care and Use Committee (IACUC) at Kunming Institute of Zoology, CAS. The animals used for SARS-CoV-2 challenge were maintained at the Animal Biosafety Level 3 (ABSL3) facility.

Protein expression and purification

The coding sequences of SARS-CoV-2 RBDs (including PT, Alpha, Beta, Gamma, Delta, Omicron sub-variants BA.1, BA.1.1, BA.2, BA.2.12.1, BA.2.75, BA.3 and BA.4/5) and nanobodies R211, S102 and R14 with a C-terminal His-tag as well as R211 with a hFc-tag were cloned into the pCAGGS vector, respectively. The recombinant plasmids were transfected into Freestyle 293F cells to express the RBD or nanobody proteins, respectively. The heavy and light chain plasmids of 74 and multispecific antibodies were cloned into the pCAGGS vector, respectively, and they were co-transfected into Freestyle 293F cells at a ratio of 1:2 to express antibody proteins. After 5 days, the supernatants were collected, and His-tagged RBD and nanobody proteins were purified by Ni affinity chromatography using a HisTrap excel 5 mL column (GE Healthcare) and hFc-tagged antibodies were purified using a Protein A 5 mL column (GE Healthcare). The proteins were further purified via gel filtration chromatography

with a Superdex 200 column (GE Healthcare).

SPR analysis

The binding affinities and kinetics between RBDs and R211 were analyzed using the BIAcore 8K (GE Healthcare) at 25 ℃ in a single-cycle mode. PBST buffer (10 mM Na_2HPO_4, 2 mM KH_2PO_4, 137 mM NaCl, 2.7 mM KCl, pH 7.4, and 0.005% (v/v) Tween 20) was used as running buffer, and RBD proteins were changed into this buffer by gel filtration before use. First, hFc-tagged R211 proteins were injected and captured on a Protein A chip (GE Healthcare) at approximately 450 response units. Serially diluted RBDs were then flowed over the surface of the chip to measure the binding response. The dissociation time of R211 from SARS-CoV-2 PT, Alpha, Beta, Gamma, Delta, BA.1, BA.1.1, BA.2, BA.2.12.1 and BA.2.75 RBDs was 600 s and from BA.4/5 RBD was 100 s. 10 mM Glycine-HCl (pH 1.5) was used to regenerate the chips. The association constant (k_a), dissociation constant (k_d) and equilibrium dissociation constant (K_D) of each pair of interactions were calculated using a 1:1 (Langmuir) binding fit model with the BIAcore 8K evaluation software.

Pseudovirus neutralization assay

VSV-ΔG-GFP-based SARS-CoV-2 PT, Alpha, Beta, Gamma, Delta, BA.1, BA.1.1, BA.2, BA.2.12.1, BA.2.75, BA.3, BA.4/5, BF.7, BQ.1, BQ.1.1, XBB, XBB.1.5, XBB.1.6, GD/1/2019, GX/P2V/2017, RaTG13, SARS-CoV and WIV1 pseudoviruses were prepared as previously described[3]. Briefly, 30 μg of the plasmids encoding viral spike (S) protein with C-terminal 18 residues deleted (S-Δ18) was transfected into HEK293T cells; 24 h later, the VSV-ΔG-G-GFP pseudoviruses were added there. After 1 h of incubation, the HEK293T cell culture medium was removed and replaced with fresh DMEM containing 10 μg/mL of anti-VSV-G antibody (I1-Hybridoma ATCC® CRL2700). After another 30 h, supernatants containing VSV-ΔG-GFP-based pseudoviruses were harvested, centrifuged and filtered through a 0.45 μm sterilized membrane filter. The pseudoviruses were then aliquoted and stored at -80 °C until use. For the neutralization assay, Vero E6 cells were seeded in 96-well plates 12 h before infection. Particularly, HEK293T-hACE2 cells were used for RaTG13 pseudovirus infection. Antibodies were 3-fold serially diluted starting from 5400, 2700, 1200, 1000,

500, 200, 100, 50, 20, 10 or 2.5 μg/mL. Then, 50 μL of the serially diluted antibodies were incubated with 50 μL of each pseudovirus at 1,000 transducing units at 37 °C for 1 h. The mixtures were then added to pre-prepared cells. After 15 h of incubation, transducing unit numbers were calculated using a CQ1 confocal image cytometer (Yokogawa). The results were analyzed using GraphPad Prism 8. Additionally, the neutralizing potencies of 74, S102 and R211 cocktail and 74, S102 and R14 cocktail in a molar ratio of 1:2:2 were also assessed against SARS-CoV-2 PT, Beta, BA.1, BA.2, BA.4/5 and XBB.

Flow cytometry assay

The S-Δ18 of SARS-CoV-2 PT, GX/P2V/2017, RaTG13, RsYN04, RshTT182, SARS-CoV, WIV1, BM48-31, BtKY72 or RpYN06 fused with green fluorescence protein (GFP) at C-terminus were expressed on the cell surface by transfecting plasmids into BHK-21 cells using PEI. After 6 h, the medium was changed to fresh DMEM supplemented with 10% FBS. 48 h later, the cells were collected and transferred to a 96-well plate (2×10^5 cells/well) for staining. Briefly, 4-fold serially diluted His-tagged R211, S102 and R14 and hFc-tagged 74 starting from 500 nM were incubated with the cells at 37°C for 30 min, respectively, with the exception of R211 with GX/P2V/2017, S102 with WIV1, R14 with SARS-CoV-2 PT and 74 with SARS-CoV, the starting concentrations of which were 0.49 nM, 125 nM, 31.25 nM and 125 nM, respectively. Subsequently, cells were washed twice and further stained with anti-His/APC antibody (Miltenyi Biotec, AB_2751870) for R211, S102 and R14 and anti-hFc/APC antibody (Biolegend, 409306) for 74 at 37°C for 30 min. After washing, the cells were analyzed using BD LSRFortessa. The results were analyzed using FlowJo V10 and GraphPad Prism 8.

Epitope competition assay

Epitope competition experiments were performed using an Octet RED96 instrument (ForteBio) at 30℃ with shaking at 1,000 rpm. Biotinylated SARS-CoV-2 PT RBD proteins were immobilized on SA biosensors (Sartorius) at 15 μg/mL. The first antibody was captured at 200 nM for 500 s and then 200 nM of the second antibody was

associated for 500 s in the presence of the first antibody. The bound antibodies were finally removed with 10 mM Glycine (pH 2.5). The results were analyzed using ForteBio Octet Data Analysis Software 9.0 and GraphPad Prism 8.

Cryo-EM sample preparation and data acquisition

For the R211/SARS-CoV-2 S complex, C-flat R2/1 (300 mesh) holey carbon grids were first glow discharged for 20 s using a Pelco easiGlow glow discharge unit and 3 μL protein was applied to the surface of the grid at a temperature of 4°C and a humidity level of 95%. Grids were then blotted for 2 s before being plunge-frozen in liquid ethane using Vitrobot Mark IV (Thermo Fisher Scientific). Grids were imaged using 300 kV Titan Krios electron microscope (Thermo Fisher Scientific) equipped with Falcon4 direct electron detector. The microscope is equipped with a GIF-Quantum energy filter (Gatan), which was used with a slit width of 10 eV. Automatic data collection was performed using EPU software. Images were recorded with Falcon4 direct electron detector operating in counting mode at pixel size of 0.84 Å. The exposure was performed with a dose rate of 15 e-/pixel/s and an accumulative dose of ~50 e-/Å2 for each image which was fractionated into 40 movie-frames. The final defocus ranges of the datasets were approximately -(1.2-2.2) μm.

Image processing and 3D reconstruction

A total of 9,348 super-resolution movies were collected and corrected for drift using MotionCorr2[4], and contrast transfer function (CTF) parameters were determined using CTF estimation in patch mode[5]. Micrographs with an estimated resolution limit worse than 5 Å were discarded in the initial screening. Blob particle picking, particle extraction and 2D classification were performed on a subset of 1,000 micrographs. Good classes were selected and subjected to template picking, which results in a total of 2,598,489 particles. After extraction and split, these particles were used in batch 2D classifications. A clean dataset with 883,179 particles from good 2D classes was selected and subjected to two rounds initial reconstruction and heterogeneous refinement. Two predominant classes showed the good structural features were

imported to Relion-3.1 for further 3D classification. After one round of 3D classification, 271,362 particles were selected and imported back to cryoSPARC[6] and obtained the structure at a 3.01 Å global resolution. Local refinement focused on the R211/SARS-CoV-2 RBD with mask could reconstitute complex structure at a 3.54 Å resolution. Local resolution estimate was performed with cryoSPARC.

Model building

The structure of the SARS-CoV-2 RBD (PDB:6M0J), was docked into the cryo-EM density maps of the R211/SARS-CoV-2 RBD complex of using CHIMERA[7]. The model was manually corrected for local fit in COOT[8] and the sequence register was updated based on alignment. The model was refined against corresponding map in real space using PHENIX[9], in which the secondary structural restraints and Ramachandran restrains were applied. The stereochemical quality of each model was assessed using MolProbity[10]. Statistics for model refinement and validation are shown in Table S1.

Live SARS-CoV-2 virus neutralization assay

The neutralizing activities of antibodies against live SARS-CoV-2 virus were determined based on the cytopathic effect (CPE). Briefly, 50 μL of 3-fold serial dilutions (starting concentration 25 μg/mL) of antibodies were incubated with an equal volume of 100 $TCID_{50}$ of live SARS-CoV-2 virus at 37°C for 1 h. The mixtures were then added to Vero E6 cells (96-well plate, 2×10^4 cells/well) and incubated for 4 days at 37°C. CPE was observed and recorded on day 5. The results were analyzed using GraphPad Prism 8. All experiments were performed in the BSL-3 facility of Kunming Institute of Zoology, CAS.

Animal protection experiments

Three- to four-week-old male Syrian hamsters were purchased from Vital River (Beijing, China) and randomly allocated to groups. All of the infected animals were housed at the ABSL-3 facility of Kunming Institute of Zoology, CAS on a 12-h light/dark cycle, with free access to food and water. In the prophylactic experiment, 15 mg/kg indicated antibodies or PBS were administered by intraperitoneal route (i.p.) 6

h before intranasal infection with 100 μL of BA.2 at 1×10^4 $TCID_{50}$. In the therapeutic experiment, animals were treated with 15 mg/kg indicated antibodies or PBS 6 h following intranasal infection with BA.2 at the same dose. All hamsters were euthanized three days post-infection, and lungs and nasal turbinates were collected for the determination of viral titers. All experiments were performed at the ABSL3 facility of Kunming Institute of Zoology, CAS.

Measurement of viral RNAs

The amounts of RNA copies per microgram RNA of lungs were determined using a quantitative real-time PCR (qRT-PCR) assay as described in our previous study[11]. In brief, Trizol Reagent (Thermo Fisher Scientific, USA) was used for homogenized tissue RNA isolation. Isolated RNAs were detected by one-step RT-PCR using a THUNDERBIRD Probe One-Step qRT-PCR kit (TOYOBO, Japan) and amplified in a BioRad CFX Real-Time PCR system. The PCR conditions were 10 min at 50 °C for reverse transcription, 60 s at 95 °C, followed by 40 cycles of 95 °C for 15 s and 60 °C for 45 s. We used the following forward (F) and reverse (R) primers and probe (P) for quantification of viral copies: genomic N gene, N-F 5'-GGGGAACTTCTCCTGCTAGAAT-3'/N-R 5'-CAGACATTTTGCTCTCAAGCTG-3', probe N-P 5'-FAM-TTGCTGCTGCTTGACAGATT-TRMRA-3'; genomic E gene, E-F 5'-ACAGGTACGTTAATAGTTAATAGCGT-3'/E-R 5'-ATATTGCAGCAGTACGCACACA-3', probe E-P 5'-FAM-ACACTAGCCATCCTTACTGCGCTTCG-TRMRA-3'; subgenomic E gene (sgE), sgE-F 5'-CGATCTCTTGTAGATCTGTTCTC-3'/sgE-R 5'-ATATTGCAGCAGTACGCACACA-3', probe sgE-P 5'-FAM-ACACTAGCCATCCTTACTGCGCTTCG-TAMRA-3'. In each run, serial dilutions of the SARS-CoV-2 RNA reference standard (National Institute of Metrology, China) were used in parallel to calculate copy numbers in each sample.

References

1 Xu, L. *et al.* COVID-19-like symptoms observed in Chinese tree shrews infected with SARS-CoV-2. *Zool. Res.* **41**, 517-526 (2020).

2 Feng, X. L. *et al.* Characteristics of replication and pathogenicity of SARS-CoV-2 Alpha and

Delta isolates. *Virol. Sin.* **37**, 804-812 (2022).

3 Zheng, A. *et al.* A binding-enhanced but enzymatic activity-eliminated human ACE2 efficiently neutralizes SARS-CoV-2 variants. *Signal Transduct Target Ther* **7**, 10 (2022).

4 Zheng, S. Q. *et al.* MotionCor2: anisotropic correction of beam-induced motion for improved cryo-electron microscopy. *Nat. Methods* **14**, 331-332 (2017).

5 Rohou, A. & Grigorieff, N. CTFFIND4: Fast and accurate defocus estimation from electron micrographs. *J Struct Biol* **192**, 216-221 (2015).

6 Punjani, A., Rubinstein, J. L., Fleet, D. J. & Brubaker, M. A. cryoSPARC: algorithms for rapid unsupervised cryo-EM structure determination. *Nat. Methods* **14**, 290-296 (2017).

7 Pettersen, E. F. *et al.* UCSF Chimera--a visualization system for exploratory research and analysis. *J. Comput. Chem.* **25**, 1605-1612 (2004).

8 Emsley, P. & Cowtan, K. Coot: model-building tools for molecular graphics. *Acta Crystallogr. D Biol. Crystallogr.* **60**, 2126-2132 (2004).

9 Adams, P. D. *et al.* PHENIX: a comprehensive Python-based system for macromolecular structure solution. *Acta Crystallogr. D Biol. Crystallogr.* **66**, 213-221 (2010).

10 Chen, V. B. *et al.* MolProbity: all-atom structure validation for macromolecular crystallography. *Acta Crystallogr. D Biol. Crystallogr.* **66**, 12-21 (2010).

11 Yu, D. *et al.* Infectivity of SARS-CoV-2 and protection against reinfection in rats. *Zool. Res.* **43**, 945-948 (2022).
